# Supplementary figures and images for: Arrayed single-gene perturbations identify drivers of human anterior neural tube closure
Source: eLife. 2026 Jul 7;14:RP108224. doi: 10.7554/eLife.108224 (PMC13341112; doi:10.7554/eLife.108224)

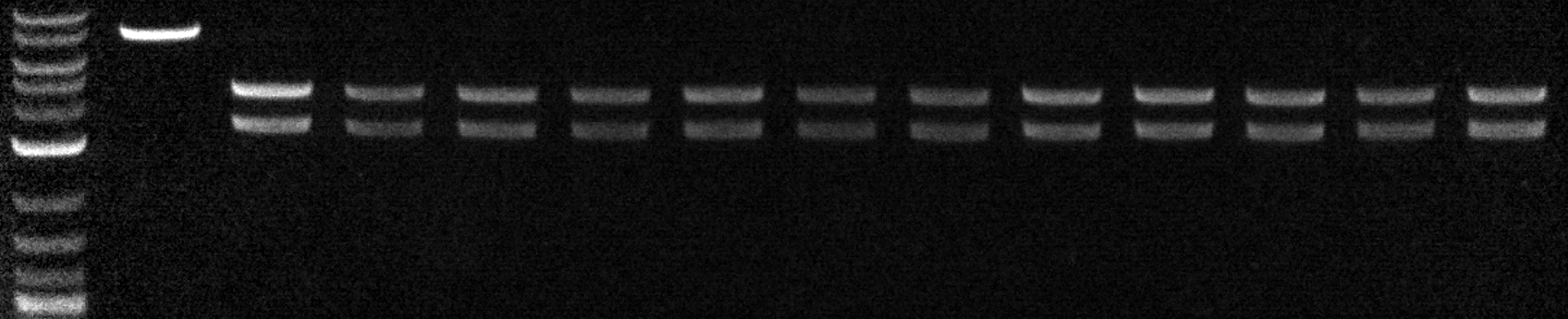

Supplement: Figure 3—figure supplement 1—source data 1. [file elife-108224-fig3-figsupp1-data1.zip › Figure3-sourcedata2/gel2.tif]

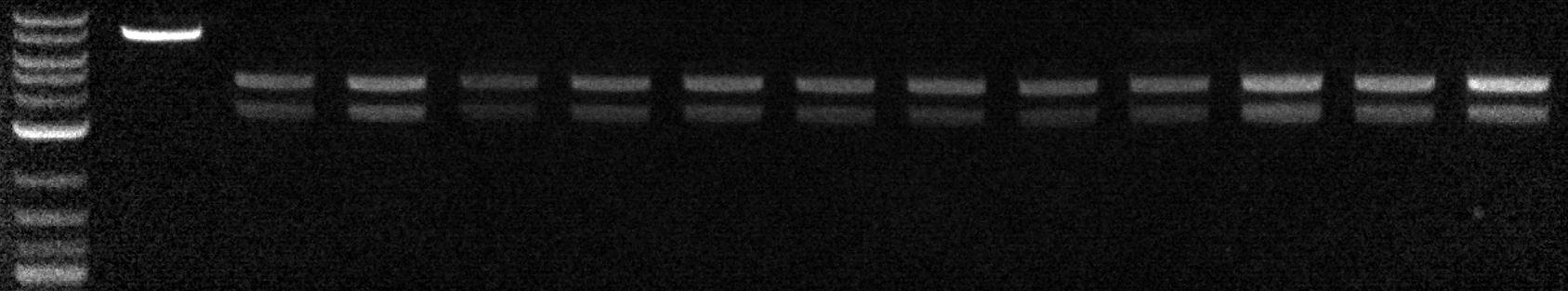

Supplement: Figure 3—figure supplement 1—source data 1. [file elife-108224-fig3-figsupp1-data1.zip › Figure3-sourcedata2/gel1.tif]

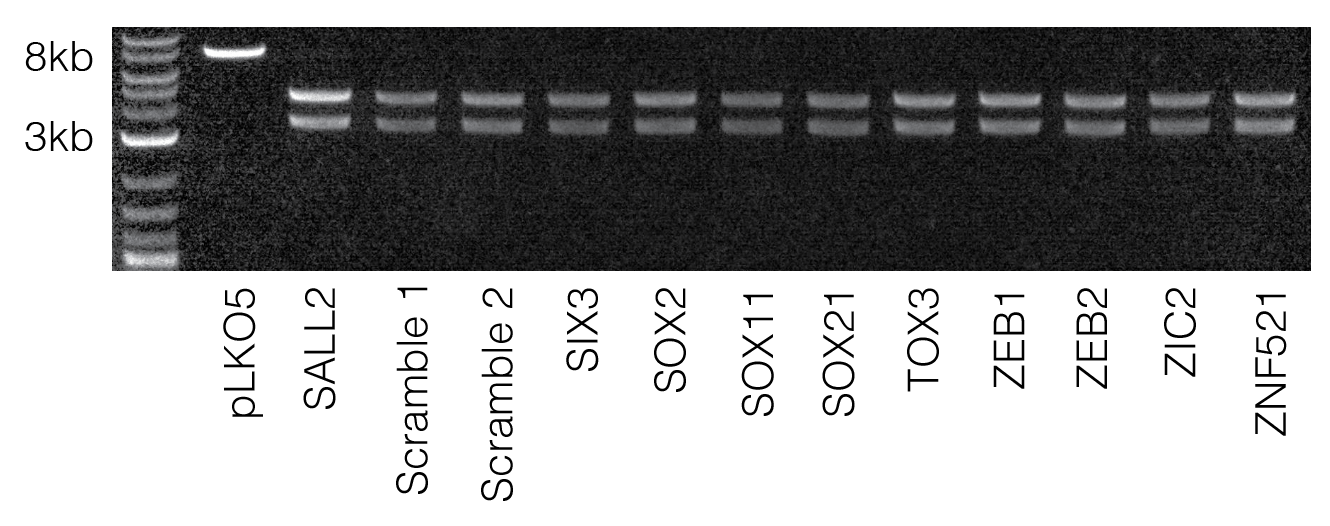

Supplement: Figure 3—figure supplement 1—source data 2. [file elife-108224-fig3-figsupp1-data2.zip › Figure3-sourcedata3/gel2.png]

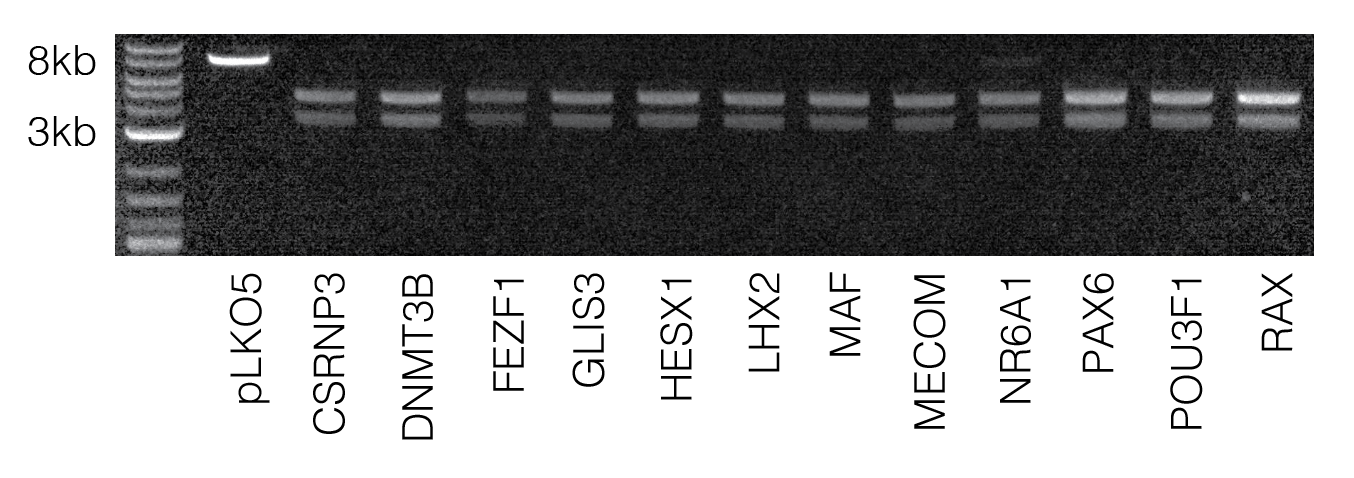

Supplement: Figure 3—figure supplement 1—source data 2. [file elife-108224-fig3-figsupp1-data2.zip › Figure3-sourcedata3/gel1.png]
