# Supplementary material for: Arrayed single-gene perturbations identify drivers of human anterior neural tube closure
Source: eLife. 2026 Jul 7;14:RP108224. doi: 10.7554/eLife.108224 (PMC13341112; doi:10.7554/eLife.108224)
Supplement: Peer review history

# Editor report: eLife Assessment

**DOI:** 10.7554/eLife.108224.3.sa0

**Authors:** Valerie Horsley

**Affiliations:** Yale University United States

**Keywords:** Exceptional, Landmark

This **landmark** study investigates how patterned human gastruloids can provide insights into neural tube closure. Using a screen, they identified positive and negative regulators and defines the epistasis among them using optimization of micro-pattern based gastruloid protocol and CRISPRi. This technical tour de force is **exceptional** and one of the first studies to reveal new knowledge on human development through embryo models.

# Referee report: Reviewer #1 (Public review)

**DOI:** 10.7554/eLife.108224.3.sa1

Summary:

This is a wonderful and landmark study in the field of human embryo modeling that uses patterned human gastruloids and conducts a functional screen on neural tube closure and identified positive and negative regulators and defines the epistasis among them.

Strengths:

This was achieved following optimization of micro-pattern based gastruloid protocol to achieve high efficiency, and then optimize was to conduct and deliver CRISPRi without disrupting the protocol. This is a technical tour de force as well as one of the first studies to reveal new knowledge on human development through embryo models which has not been done before.

Weaknesses:

A minor one. One can never find out if findings in human embryo models can be in vitro revalidated in humans in vivo for obvious and justified ethical reasons. However, the authors indicate that in the "limitations of study" section.

Comments on revisions:

The authors have adequately addressed all comments raised.

# Author comment: Author response

**DOI:** 10.7554/eLife.108224.3.sa2

**Authors:** Roya E Huang, Giridhar M Anand, Heitor C Megale, Jason Chen, Chudi Abraham-Igwe, Sharad Ramanathan

**Affiliations:** Harvard University Cambridge United States; Harvard University Cambridge United States; Harvard University Cambridge United States; Harvard University Cambridge United States; Harvard University Cambridge United States; Harvard University Cambridge United States

The following is the authors’ response to the original reviews.

**Public Reviews:**

**Reviewer #1 (Public review):**

Summary:

This is a wonderful and landmark study in the field of human embryo modeling. It uses patterned human gastruloids and conducts a functional screen on neural tube closure, and identifies positive and negative regulators, and defines the epistasis among them.

Strengths:

The above was achieved following optimization of the micro-pattern-based gastruloid protocol to achieve high efficiency, and then optimized to conduct and deliver CRISPRi without disrupting the protocol. This is a technical tour de force as well as one of the first studies to reveal new knowledge on human development through embryo models, which has not been done before.

The manuscript is very solid and well-written. The figures are clear, elegant, and meaningful. The conclusions are fully supported by the data shown. The methods are well-detailed, which is very important for such a study.

Thank you for this feedback! We are excited for the possibilities of this method to discover genes required for various morphogenetic processes associated with human embryonic development.

Weaknesses:

This reviewer did not identify any meaningful, major, or minor caveats that need addressing or correcting.

A minor weakness is that one can never find out if the findings in human embryo models can be in vitro revalidated in humans in vivo. This is for obvious and justified ethical reasons. However, the authors acknowledge this point in the section of the manuscript detailing the limitations of their study.

**Reviewer #2 (Public review):**

Summary:

This manuscript is a technical report on a new model of early neurogenesis, coupled to a novel platform for genetic screens. The model is more faithful than others published to date, and the screening platform is an advance over existing ones in terms of speed and throughput.

Thank you for this feedback! We agree that the robust symmetry breaking observed in our model, the comparisons to the human embryo in our cell type analysis, and the ability to conduct large-scale genetic screens represent advancements in the modeling of human neural tube closure that may be built upon in the future.

Strengths:

It is novel and useful.

Weaknesses:

The novelty of the results is limited in terms of biology, mainly a proof of concept of the platform and a very good demonstration of the hierarchical interactions of the top regulators of GRNs.

The value of the manuscript could be enhanced in two ways:

(1) by showing its versatility and transforming the level of neural tube to midbrain and hindbrain, and looking at the transcriptional hierarchies there.

We thank the reviewer for this valuable suggestion and will keep this in mind for future work. As accurate answers to this question would require the development of robust midbrain and hindbrain organoid models, we believe that this question is outside the scope of the present work.

(2) by relating the patterning of the organoids to the situation in vivo, in particular with the information in reference 49. The authors make a statement "To compare our findings with in vivo gene expression patterns, we applied the same approach to published scRNA-seq data from 4-week-old human embryos at the neurula stage" but it would be good to have a more nuanced reference: what stage, what genes are missing, what do they add to the information in that reference?

We agree that a more comprehensive comparison of in vitro and in vivo data would add value to the study. We have added an analysis of the human Week 3 data, as neurulation occurs between Weeks 3 and 4 of human embryogenesis (new Figure 1F). We see our in vitro cell types in both datasets. We also included volcano plots in our supplementary figure to show major differences in gene expression (new Figure S1G). Somewhat surprisingly, embryo samples show higher expression of hemoglobin subunits and other hypoxia-related genes than organoids do, which may indicate hypoxic stress during sample handling during ex vivo experimentation (Schelshortn, et al., 2008) or alternatively, reflect differences in the metabolic environment between embryos and organoids. We did not find any differences would have affected our transcription factor candidate selection.

**Recommendations for the authors:**

**Reviewing Editor Comments:**

The reviewers were very enthusiastic about the work and provided suggestions for textual changes that will clarify the figures, methods, and results for readers.

**Reviewer #2 (Recommendations for the authors):**

(1) In Figure 1:

(a) What is the orientation of the images in 1C?

We have specified in the text and figure legend that this is a top-down view of an outer organoid.

In this panel, what is the problem with ZO-1 in D4?

We believe this is non-specific staining of dead cells that shed into the lumen during folding and closure. We have added this interpretation to the figure legend and added two supplementary time lapse videos (new Supplementary Video 1 and new Supplementary Video 2) of organoid closure that show dead cells being shed into the lumen as support to this interpretation.

(b) What is the three-dimensional organization of these structures, if any? Or are they two-dimensional? In a way, this also refers to 1C.

We have clarified in the text and figure legend that these organoids are three dimensional, and that Fig. 1B-C are top-down views.

(c) Why can't we see FOXG1 amidst the markers forebrain? This is a very characteristic one.

We see sparse *FOXG1* expression in the human embryo samples at Week 4 (new Figure 1F), which may indicate that *FOXG1* expression is upregulated later in the human embryo, after neural tube closure. We do see high levels of other fore brain associated transcription factors by this time however, including *OTX2*, *LHX2*, and *SIX3*.

(d) The Figure 1 legend needs to be clear about the issues raised here.

We have updated the Figure 1 legend to address these points.

(2) Figure 2, could they explain in the text better how they organize the ML gene expression? What are their criteria?

We thank the reviewer for catching this critical omission. We have added details of our medio lateral axis generation to the Methods section under “Single cell RNA sequencing analysis.”

(3) Explain how and why the 77 genes were picked up?

We have clarified at our first mention of 77 genes that this is a subset of our original 78 candidate genes, which were selected as described in the text (last paragraph in the results section) “Identifying transcription factor candidates for regulation of anterior neurulation”. We have added a line in the Methods section that we were unable to clone a functional guide plasmid against one our candidates (NR6A1).

(4) The authors mention the value of the geometry and the mechanics in neural tube closure, but they make no attempt to unravel these inputs, or at least the genes, from their screen, associated with them.

We have rewritten this discussion of the literature to emphasize the active role of the neural ectoderm compared to the surface ectoderm, in order to justify the genetic analysis of the neural ectoderm rather than the surface ectoderm. We have clarified that our goal is to find upstream developmental drivers (transcription factors) of folding and closure, rather than investigate mechanical mechanisms of this process.
